# Oxygen systems strengthening as an intervention to prevent childhood deaths due to pneumonia in low-resource settings: systematic review, meta-analysis and cost-effectiveness

**DOI:** 10.1136/bmjgh-2021-007468

**Published:** 2021-12-20

**Authors:** Felix Lam, Angela Stegmuller, Victoria B Chou, Hamish R Graham

**Affiliations:** 1 Clinton Health Access Initiative, Boston, Massachusetts, USA; 2 International Health, Johns Hopkins University Bloomberg School of Public Health, Baltimore, Maryland, USA; 3 Centre for International Child Health, University of Melbourne, MCRI, Royal Children's Hospital, Parkville, Victoria, Australia; 4 Department of Paediatrics, University College Hospital Ibadan, Ibadan, Nigeria

**Keywords:** pneumonia, systematic review, child health, health economics

## Abstract

**Objectives:**

Increasing access to oxygen services may improve outcomes among children with pneumonia living in low-resource settings. We conducted a systematic review to estimate the impact and cost-effectiveness of strengthening oxygen services in low-income and middle-income countries with the objective of including oxygen as an intervention in the Lives Saved Tool.

**Design:**

We searched EMBASE and PubMed on 31 March 2021 using keywords and MeSH terms related to ‘oxygen’, ‘pneumonia’ and ‘child’ without restrictions on language or date. The risk of bias was assessed for all included studies using the quality assessment tool for quantitative studies, and we assessed the overall certainty of the evidence using Grading of Recommendations, Assessment, Development and Evaluations. Meta-analysis methods using random effects with inverse-variance weights was used to calculate a pooled OR and 95% CIs. Programme cost data were extracted from full study reports and correspondence with study authors, and we estimated cost-effectiveness in US dollar per disability-adjusted life-year (DALY) averted.

**Results:**

Our search identified 665 studies. Four studies were included in the review involving 75 hospitals and 34 485 study participants. We calculated a pooled OR of 0.52 (95% CI 0.39 to 0.70) in favour of oxygen systems reducing childhood pneumonia mortality. The median cost-effectiveness of oxygen systems strengthening was $US62 per DALY averted (range: US$44–US$225). We graded the risk of bias as moderate and the overall certainty of the evidence as low due to the non-randomised design of the studies.

**Conclusion:**

Our findings suggest that strengthening oxygen systems is likely to reduce hospital-based pneumonia mortality and may be cost-effective in low-resource settings. Additional implementation trials using more rigorous designs are needed to strengthen the certainty in the effect estimate.

**Key questions**

**What is already known?**

- WHO recommends oxygen therapy for management of hypoxaemia in low-resource settings.
- Oxygen can be feasibly introduced and used in low-resource settings for management of hypoxaemia and individual studies have found mortality reductions, though with variable results.
- We know little about the cost-effectiveness of investments to increase oxygen utilisation for pneumonia.

**What are the new findings?**

- Interventions to strengthen oxygen systems are likely to reduce pneumonia mortality and these interventions are cost-effective.
- There are few published studies examining the effect of oxygen systems on pneumonia mortality in children; all of the studies used an observational, non-randomised design resulting in moderate risk of bias and low certainty in the overall evidence.

**What do the new findings imply?**

- Global health should prioritise oxygen systems strengthening as an intervention to address childhood pneumonia deaths in low-resource settings.
- Additional research using more rigorous designs is needed to strengthen the certainty in the estimate of effect.

## Background

Pneumonia is the leading infectious cause of mortality among children under-5 in low-income and middle-income countries (LMICs).^1^ Children with pneumonia are at risk for developing hypoxaemia, or low levels of oxygen in the blood, which greatly increases the likelihood of death.^2^ Oxygen is an important intervention for patients with hypoxaemia, and therefore, children with pneumonia could greatly benefit from increased access to this life-saving therapy. While oxygen is included as one of the interventions in the Global Action Plan for Pneumonia and Diarrhoea, it has received less attention than other interventions, such as vaccines, breast feeding, indoor air pollution reduction and antibiotics, as evidenced by the lack of global investment and indicators to track oxygen scale-up.^3^ The lack of prioritisation may be due in part to perceptions that investment in oxygen systems are expensive.

In this paper, we aim to review evidence on the effectiveness of strengthening oxygen systems on mortality for children with pneumonia with the purpose of populating estimates in the Lives Saved Tool (LiST) and estimating the potential public health benefits of increased access to oxygen therapy. The LiST is a model that estimates the impact of scaling up on maternal, newborn and child health, and nutrition interventions in LMICs.^4^ LiST is often used for strategic planning, programme evaluation, and advocacy by governments, donors and international organisations, and inclusion of oxygen in LiST could support efforts to prioritise it within the context of other child health interventions.^5^ A previous review completed by Catto *et al* conservatively estimated that improving oxygen systems could reduce child pneumonia mortality by 20%, saving 68 000–122 000 child lives annually. However, the authors were hampered by lack of effectiveness data from multiple contexts and the resulting evidence was insufficient for inclusion into LiST.^6^ In this review, we build on this previous work to establish the effectiveness and cost-effectiveness of strengthening oxygen systems on childhood pneumonia mortality in low-ncome and middle-income countries.

## Methods

### Aims and objectives

The aim of the study was to estimate the impact and cost-effectiveness of improved oxygen systems on pneumonia mortality in children under-5 compared with usual care with the objective of including oxygen as an intervention in the LiST.

### Search strategy

We searched two databases (EMBASE and PubMed) for peer-reviewed literature using keywords and MeSH terms related to ‘oxygen’, ‘pneumonia’ and ‘child’ without limitation on language or date. We identified search terms from previous reports and literature reviews, with help from a public health informationist, and tested them to ensure known eligible studies were retrieved. Details of the search strategy and databases searched are presented in online supplemental file 1. AS conducted the search on 31 March 2021. We also reviewed reference lists of included studies and the previous systematic review and contacted corresponding authors and experts in child pneumonia and/or oxygen therapy to identify additional studies not located by the database search.

10.1136/bmjgh-2021-007468.supp1 Supplementary data

Results from the searches were exported to Covidence (Veritas Health Innovation, Melbourne, Australia) for managing the review and data extraction. AS and VBC independently screened the abstracts of each study. Studies were included if the study involved children aged 1–59 months with pneumonia, had a comparator or control arm, and included the provision of both oxygen therapy and pulse oximetry as part of the intervention. We excluded studies that were conducted in the intensive care unit, included mechanical ventilation, or studied advanced delivery methods such as continuous positive airway pressure (CPAP) or bubble CPAP. We excluded studies exclusively focused on neonatal populations. If AS and VBC had conflicting decisions on a study, FL reviewed the abstract and provided a final decision. AS and FL conducted a full-text review of studies passing the abstract screening. AS and FL discussed any conflicting reviews and made a joint final decision.

### Data extraction

AS and FL extracted study data using a standardised form in Covidence. Key variables extracted include publication details, timing of the study, description of the study population and any subgroups, description of the intervention and context, number of participants and number who died by study arm and mortality impact estimate. Where multiple analyses were reported (eg, on different subpopulations or at different stages of intervention), we first looked for estimates that precisely met our study population (ie, hospitalised children under-5 with pneumonia). If the study included our population of interest, but did not present results specifically for our study population (ie, all paediatric patients instead of under-5), we contacted study authors for clarification or request for reanalysis.

Detailed cost data were also extracted from the full reports, including data on equipment, installation and educational activities, maintenance and ongoing support. Where not published, we contacted study authors to gather data on the costs of programme implementation.

FL assessed risk of bias for all included studies using the quality assessment tool for quantitative studies.^7^ This tool enables structured evaluation of potential bias in study design, participant selection, confounding, blinding, data collection methods, and withdrawals and drop-outs, has been validated against the Cochrane risk of bias tool and is applicable to all interventional studies.^7 8^

### Data analysis

We present summary details on all studies included in qualitative synthesis, including details on the study design, population, intervention details and context. We included all studies with comparable outcome data in quantitative analysis using generic inverse variance with random effects to calculate a pooled effect estimate with 95% CIs using Review Manager (RevMan V.5.4) (The Cochrane Collaboration, 2020). We expressed the intervention effect as ORs comparing the intervention group to the control group and reported the individual and pooled effect sizes in tables and forest plots. We visually depicted heterogeneity between studies in a forest plot and discussed this heterogeneity with respect to the study context and interventional components in qualitative synthesis but did not attempt quantitative subgroup analysis. To assess outcome reporting bias, FL reviewed study protocols and published reports, comparing the outcomes specified in the protocol (or the Methods section of report if protocol not available) with the outcomes reported in the corresponding report. To assess the certainty of these estimates FL considered each of the Grading of Recommendations, Assessment, Development and Evaluations (GRADE) domains (risk of bias, imprecision, inconsistency, indirectness, publication bias, magnitude of effect and effects of residual confounding) and then gave an overall confidence score of very low, low, moderate or high.^9^

We calculated cost-effectiveness as disability-adjusted life-years (DALYs) averted per dollar and deaths averted per dollar. Cost data were first categorised into three groups: equipment costs (including freight and customs), implementation (ie, training, installation, maintenance), and solar power where relevant. All study costs were adjusted for inflation and converted to US dollar in the year 2000. The year for study costs were taken as the midpoint of the study.

We estimated number of deaths averted in each study in two steps. We first constructed a counterfactual by dividing the number of pneumonia deaths in the intervention arm by the intervention effect estimate for each study. Then, we took the difference between the observed number of deaths in the intervention arm of the study and the calculated counterfactual estimate to estimate the number of pneumonia deaths averted. To estimate the number of DALYS averted, we multiplied the number of deaths averted by 33, corresponding to the number of DALYs lost due to a death in infancy.^10^ As all studies did not include solar power equipment as part of the intervention package, we estimated cost-effectiveness of strengthening oxygen systems without solar costs using all studies and cost-effectiveness with solar for only studies that included it.

Cost-effectiveness calculations were conducted in Google Sheets (Alphabet, Mountain View, California, USA).

## Results

### Search results

**Figure 1:**
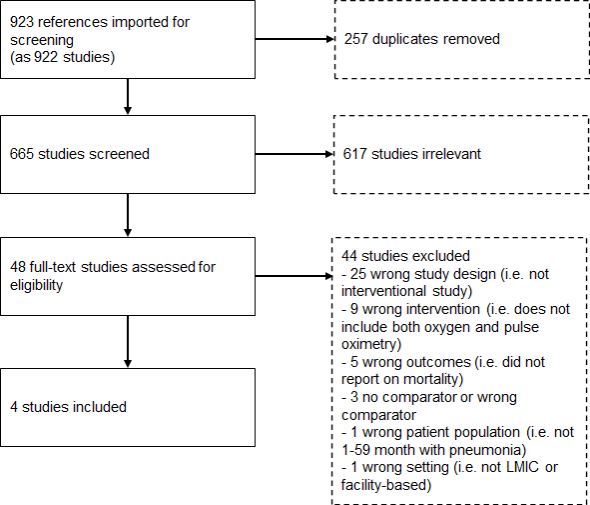
Flow diagram of search results. LMIC, low-and-middle income country.

Figure 1 presents results from the search results. After removing duplicates, we identified 665 studies for abstract review. Forty-eight studies were included for full text review, and four studies met all criteria for inclusion. No additional studies were identified through expert consultation, and experts reaffirmed that the four studies were the only ones they were aware of.

### Study descriptive summaries

Table 1 provides an overview of the four studies included in the review. The studies reviewed included 75 hospitals and 34 485 study participants. Duke *et al* conducted two non-randomised pre–post prospective oxygen intervention studies in hospitals in Papua New Guinea involving 5 hospitals (2005–2007) and 38 rural health facilities (2015–2017), respectively.^11 12^ Gray *et al* conducted a non-randomised controlled prospective evaluation of oxygen systems in 20 (10 intervention, 10 control) hospitals in Laos (2011–2013).^13^ Graham *et al* conducted a stepped-wedge cluster-randomised trial in 12 hospitals in Southwest Nigeria (2015–2017) using the stepped wedge design to evaluate pulse oximetry alone compared with full oxygen system and mixed-effects regression to compare against preintervention mortality rates.^14^

**Table 1:** Summary description of included studies

Study	Country	Study setting	Study design	Study period	No of patients (deaths)	Effect estimate: OR (95% CI)	Quality assessment rating
Duke^11^	Papua New Guinea	5 hospitals (3 in highland, 1 coastal and 1 inland)	Prospective before-and-after controlled study	2005–2007	11 291 (489)	0.64 (0.52 to 0.78)	Moderate
Gray^13^	Lao PDR	20 district hospitals	Prospective before-and-after controlled study	2011–2013	1403 (25)	0.32 (0.13 to 0.80)	Moderate
Graham^14^	Nigeria	12 secondary level hospitals in Southwest Nigeria (Oyo, Ondo, Ogun, and Osun states)	Stepped-wedge cluster randomised trial with a prospective before-and-after extended analysis	2015–2017	2858 (195)	0.46 (0.23 to 0.92)	Strong
Duke^12^	Papua New Guinea	38 rural hospitals	Prospective before-and-after controlled study	2015–2017	18 933 (530)	0.47 (0.39 to 0.57)	Moderate

#### Context

The first Papua New Guinea study involved four tertiary (provincial) hospitals and one secondary (district) hospital in highland and lowland areas of Papua New Guinea, each admitting 600–2500 children annually.^11^ The Lao PDR and Nigerian studies both focused on secondary (district) hospitals that admitted around 50–2500 (median ~350) children annually.^13 14^ The Lao PDR study involved 20 secondary (district) hospitals (10 intervention, 10 control) distributed across Northern and Southern provinces, representing different climates and disease patterns. The Nigeria study involved 12 secondary level facilities distributed across four states in malaria-endemic south-west Nigeria. The second Papua New Guinea study included 26 primary (health centre) and 12 secondary (district/rural) hospitals, mostly located in remote areas of the highlands and admitting a median 65 (range 0–485) and 375 (range 61–1592) children annually.^12^

All studies focused activities and evaluation on children, with a particular focus on children under 5 years of age admitted with pneumonia. However, the oxygen systems introduced to facilities served broader newborn, child, and adolescent populations, and those installed in Lao PDR and the smaller facilities in Papua New Guinea also served adults.

#### Intervention

The improved oxygen systems introduced in all four included studies involved (1) equipment, including oxygen concentrators and handheld pulse oximeters, (2) educational activities for healthcare workers and biomedical engineers/technicians (typically conducted on-site), (3) some degree of ongoing support and supervision; and (4) were implemented using quality improvement approaches (eg, problem solving teams, audit and feedback). However, the specific activities within these core components varied considerably (table 2). The three studies from Papua New Guinea and Lao PDR delivered their oxygen intervention as part of a comprehensive educational programme on hospital care for children, while the Nigeria study focused training more narrowly on oxygen and pneumonia. For example, the Papua New Guinea programme described by Duke included a 5-day comprehensive child health training module delivered by visiting paediatricians at each hospital,^12 15^ while the Nigeria programme used half-day workshops focused on oximetry and oxygen.^16 17^ All programmes used quality improvement strategies (eg, problem solving teams, audit and feedback) and included follow-up supervisory and re-educational visits. All programmes used concentrator-based oxygen systems and followed similar design and installation procedures and used the same consultant for senior engineering support. The two most recent programmes included solar power provision to answer implementation questions about how to provide oxygen reliably in small and remote facilities without reliable power.

**Table 2:** Description of interventions of included studies

Study	Intervention components
Duke^11^	**Equipment and maintenance** Technical specifications of eight different oxygen concentrators were compared for suitability of use in children’s ward Fifteen AirSep oxygen concentrators (5 Elite and 10 Intensity models) were procured, distributed, commissioned and installed across the 5 hospitals One handheld pulse oximeter and several replacement probes procured for each hospital Flow splitters, tubing and nasal prongs for various child age groups procured and a regimen developed for cleaning, testing and reusing accessories At least one oxygen cylinder available as back-up Follow-up technical equipment evaluations conducted at 14 and 28 months after installation **Capacity building** Hospital engineers, clinicians and nurses involved in commissioning and installation of equipment as means of training Multiple didactic and small group hands-on trainings were also conducted to cover all clinical and technical staff **Leadership and oversight** Multidisciplinary national oxygen team consisting of paediatricians and biomedical engineers led implementation and conducted regular visits to facilities **Evaluations and assessments** Baseline assessments of facility infrastructure, power requirements, personnel, patient capacity and availability of existing oxygen equipment and other healthcare commodities Preintervention and postintervention data extracted from child ward admission books to evaluate case-fatality rates Prospectively collected all costs related to implementing the programme, including equipment, consumables, training, supervision, repairs and maintenance, and assessments
Gray^13^	**Equipment and maintenance** Approximately four Airsep VisionAire concentrators and comprehensive set of spare parts procured for each hospital One Bitmos tabletop pulse oximeter and 12 replacement probes of various sizes procured for each hospital Flow splitters, tubing, prongs, oxygen analysers and installation materials Multidisciplinary team from MOH, engineers and clinicians visited each hospital for 1 week to check the equipment, instal the oxygen system, and provide training on its use and maintenance Additional training sessions were conducted for engineers and technicians at central, provincial and district levels on installation and servicing Repairs made to one-third (of concentrators after 1 year. Seven failed after 2 years **Capacity building** Lao-specific training materials such as videos, guidelines, lectures and case-based teaching adapted from the WHO Pocketbook training were developed The WHO publication ‘The Clinical Use of Oxygen: Guidelines for healthcare workers, hospital engineers and managers.’ was translated into Lao language A Digital Video Disc (DVD) was produced using the five-part oxygen therapy video from the WHO Pocketbook of Hospital Care for Children training compact disc (CD) Laminated one-page documents were produced in Lao language to support the correct use of the oxygen equipment Practical sessions included using oximeters, nasal prongs, oxygen masks and catheters with dummies and guided examinations of patients with respiratory diseases Clinical training was provided over 2 days at each of the 10 intervention hospitals **Financial** Hospitals decided to make oxygen from concentrators freely available to all patients **Leadership and oversight** Lao National Oxygen Team consisting of staff from the MOH and Medical Products Supply Centre, national clinicians, provincial and district health staff, and international staff from the WHO and Centre for International Child Health at University of Melbourne Supervision visits by coordinators (at 3, 12 and 24 months) **Evaluations and assessments** Preintervention and postintervention evaluation using retrospective data collection using a standardised data abstraction form for medical records Prospective data collection on all patients who receive oxygen at intervention and control hospitals throughout the duration of the project Routine hospital data were collected during the intervention including number of admissions, admission diagnosis, oxygen use and cost
Graham^14^	**Equipment and maintenance** Lifebox pulse oximeters and training introduced to all hospitals prior to full oxygen system strengthening interventions Oxygen concentrators (Airsep Elite 5LPM), tubing and delivery devices, and maintenance materials were installed collaboratively by project and hospital technicians Solar-power systems with battery storage and/or petrol generators installed Hospital technicians and clinical staff trained on basic maintenance and given responsibility for various aspects of weekly and quarterly equipment checks and preventive maintenance Ongoing support from project team to assist with troubleshooting and repairs **Capacity building** Clinical training based on the WHO guidelines for Clinical Use of Oxygen in Children and WHO Hospital Care for Children Local healthcare workers, with support from project team, were trained as Master Trainers and led training sessions for their colleagues. Encouraged to do additional training for new and rotating staff. Initial clinical training conducted at hospitals, using practical, group-discussion based educational methods over 3–4 hour sessions. Hospital technicians trained at a central 3-day workshop, led by project staff and experienced UK-based engineer, and were involved in all aspects of equipment testing, installation, maintenance and repair. Wall charts, checklists and quick summary guidelines disseminated **Leadership and oversight** Oxygen Implementation Project team worked with hospital administrators to implement the programme, with governance support from federal and state health agencies. Project team visited health facilities every 3 months to provide supportive supervision, feedback, and collect user feedback. Quality improvement approach taken to strengthen project implementation using multidisciplinary hospital oxygen teams. **Evaluations and assessments** Unblinded, stepped-wedge cluster-randomised trial design taken to evaluate primary outcome of mortality between pulse oximetry alone arm to full oxygen system arm Retrospective admissions and discharge register data collected for extended analysis comparing preintervention to postintervention arms Mixed-methods design used to collect both quantitative and qualitative data on clinical and implementation outcomes
Duke^12^	**Equipment and maintenance** Design and installation of solar power system including battery backup system for 3 days Airsep Elite 5 L/min concentrators (two or three concentrators per facility) and Lifebox pulse oximeters Project teams spent 2–3 days at each facility to instal solar system and commission oxygen equipment Healthcare workers trained to conduct preventative maintenance and monitoring of equipment performance using Maxtec O2 analysers Province and district technicians and engineers provided spare parts and trained on repair and maintenance **Capacity building** Curriculum based on the WHO guidelines for Clinical Use of Oxygen in Children and WHO Hospital Care for Children Clinical and technical content delivered through 5-day workshop-based training sessions that include direct facilitator and peer-to-peer teaching modalities Follow-up site visits used to reinforce both clinical and technical skills and knowledge **Leadership and oversight** Continuous quality improvement approach taken by provincial supervisory teams consisting of a paediatrician and a technician conducting site reviews every 4–6 months. Visits included on-site training, data collection and troubleshooting of problems identified and feedback given to facility and provincial staff **Evaluations and assessments** Health facility admission and discharge registers were reviewed, and mortality rates estimated between preintervention and postintervention period

### Risk of bias assessment

Based on the design of the studies, we rated the quality of three studies (Duke, Grayand Duke) as moderate and one study as strong (Graham). The three studies were rated as moderate due to having weaker methods in controlling for confounders. All three studies used prospective before-and-after evaluation designs and relied on patient admission and discharge registers to measure mortality rates and with little or no additional data used to control for differences in admission patterns in the preintervention and postintervention periods. Details of the risk of bias assessment is presented in online supplemental file 2.

10.1136/bmjgh-2021-007468.supp2 Supplementary data

### Outcome: under-5 pneumonia mortality

**Figure 2:**
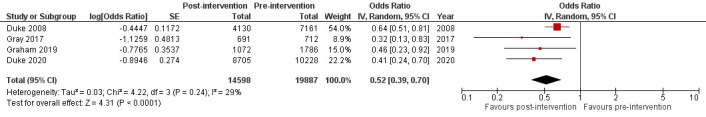
Meta-analysis results and forest plot for under-5 pneumonia mortality.

Pooled analysis of the four studies found OR 0.52 (95% CI 0.39 to 0.70) for the odds of under-5 pneumonia death comparing improved oxygen systems to standard care (figure 2). Individually, all studies found a reduction in pneumonia mortality when oxygen systems were strengthened with ORs ranging from 0.32 (95% CI 0.13 to 0.83) to 0.64 (95% CI 0.51 to 0.81). Pooled under-5 pneumonia mortality rates reduced from 4.3% to 2.6% following oxygen system strengthening, corresponding to 20 fewer deaths per 1000 cases (from 25 fewer to 14 fewer). Given the general homogeneity in study quality and outcomes, and the low number of studies, we did not conduct subgroup or sensitivity analysis. Using the GRADE, we assessed the overall certainty of the evidence as low due to the observational design of the studies (table 3).

**Table 3:** GRADE assessment of included studies

Participants (studies)	Risk of bias	Inconsistency	Indirectness	Imprecision	Publication bias	Magnitude of effect	Effects of residual confounding	Overall certainty of evidence	Study event rates (%)		Relative effect (95% CI)	Anticipated absolute effects	
									Risk of death prior to oxygen systems strengthening	Risk of death after oxygen systems strengthening		Risk of death prior to oxygen systems strengthening	Risk difference after oxygen systems strengthening
34 485 (4 observational studies)	Not serious	Not serious	Not serious	Not serious	No	Not large	No	Low	860/19887 (4.3%)	379/14598 (2.6%)	OR 0.52 (0.39 to 0.70)	43 per 1000	20 fewer per 1000 (from 25 fewer to 14 fewer)

### Outcome: under-5 all-cause mortality

**Figure 3:**
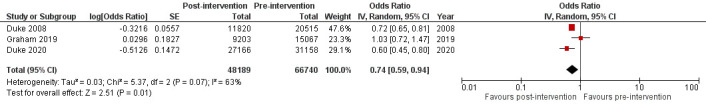
Meta-analysis results and forest plot for paediatric all-cause mortality.

Three studies —Duke and Duke in Papua New Guinea and Graham in Nigeria—reported all-cause mortality among paediatric patients admitted to the study facilities. All-cause mortality results from the first study in Papua New Guinea were reported in a separate review.^18^ The pooled analysis of the studies found OR=0.74 (95% CI 0.59 to 0.94) for the odds of under-5 death comparing improved oxygen systems to standard care (figure 3). Both studies in Papua New Guinea individually found statistically significant differences between the postintervention and preintervention periods of the studies. The odds of mortality in paediatric patients in the period after oxygen systems strengthening relative to the pre-intervention period were 0.72 (95% CI 0.65 to 0.81) in Duke and 0.60 (95% CI 0.45 to 0.80) in Duke. Results from Nigeria did not find a reduction in all-cause paediatric mortality (OR 1.03, 95% CI 0.72 to 1.47).

### Cost-effectiveness

Table 4 presents programme costs per study facility adjusted to USD in the year 2000. Graham and Duke had the highest per study facility costs –US$57 540 and US$42 432, respectively—due to the costs of solar systems which were not part of the programmes in Duke—US$21 924 per facility—or Gray—US$9448 per facility. Excluding the costs of the solar systems, the programme costs for Graham and Duke were US$19 020 and US$12 912 per facility. The relative costs of oxygen system equipment (including spare parts, ancillary supplies such as nasal prongs, and shipping) accounted for most non-solar programme costs—between 65% and 73%—and implementation costs, such as installation, training and monitoring, were 23%–35%.

**Table 4:** Programme costs (in USD in the year 2000)

Study	No of study facilities	Total programme costs				Per facility costs			
		Oxygen equipment and supplies	Implementation	Solar	Total	Oxygen equipment and supplies	Implementation	Solar	Total
Duke^11^	5	US$71 731	US$37 890	N/A	US$109 620	US$14 346	US$7578	N/A	US$21 924
Gray^13^	10	US$62 977	US$31 500	N/A	US$94 477	US$6298	US$3150	N/A	US$9448
Graham^14^	12	US$167 040	US$61 200	US$462 240	US$690 480	US$13 920	US$5100	US$38 520	US$57 540
Duke^12^	38	US$320 720	US$169 920	US$1 121 760	US$1 612 400	US$8440	US$4472	US$29 520	US$42 432

N/A, not available.

Table 5 presents the results of the cost-effectiveness calculations. Across all the studies, we estimate 410 under-5 pneumonia deaths were averted during programme implementation in the studies and approximately 13 526 DALYs averted. We estimate the median cost-effectiveness of strengthening oxygen systems (without solar costs) is US$68 per DALY averted (range: US$44–US$225). For the two studies which included costs of solar power equipment, the cost-effectiveness ranges from US$205 to US$222 per DALY averted. When considering the two Papua New Guinea studies with paediatric all-cause mortality results, we estimate the cost-effectiveness of oxygen systems ranges between US$18 and US$26 per DALY averted. The study in Nigeria did not find a reduction in paediatric all-cause mortality so a cost-effectiveness estimate could not be estimated.

**Table 5:** Cost-effectiveness estimates

Study	OR of postintervention to preintervention	Observed deaths	Estimated counterfactual deaths	Estimated deaths averted	DALYs averted	Cost per DALY averted (without solar)	Cost per DALY averted (with solar)
Under-5 pneumonia mortality							
Duke^11^	0.64	133	208	75	2469	US$44	N/A
Gray^13^	0.32	6	19	13	421	US$225	N/A
Graham^14^	0.46	87	189	102	3370	US$68	$205
Duke^12^	0.41	153	373	220	7266	US$68	$222
Paediatric all-cause mortality							
Duke^11^	0.72	481	668	187	6173	US$18	N/A
Duke^12^	0.60	867	1445	578	19 074	US$26	$85

DALYs, disability-adjusted life-years; N/A, not available.

## Discussion

Oxygen systems are an essential service for hospital care of children and adults but have not been recognised as a priority until the global COVID-19 pandemic. While oxygen is indicated from a wide variety of acute conditions and essential for safe anaesthesia and surgery, it is particularly critical for the care of children with severe pneumonia where hypoxaemia is common and deadly.^19 20^ Recent updates to global pneumonia strategies have included oxygen as a priority, but planning and investment cases have been hampered by lack of consensus on the effectiveness and cost-effectiveness of improving oxygen systems.

Our findings suggest that strengthening oxygen systems could reduce hospital-based pneumonia deaths by nearly half and hospital-based paediatric deaths overall by a quarter. One previous review of oxygen for pneumonia in LMICs was conducted by Catto *et al*.^6^ At the time of the study’s publication, only one of the studies included in this review was published. Therefore, Catto *et al* used the Child Health and Nutrition Research Initiative framework to evaluate the effectiveness of oxygen and other dimensions such as feasibility and sustainability. They found the median mortality reduction estimated by experts was 20% (IQR: 10%–35%, min. 0%, max. 50%). Our results fall in the higher end of the estimates found by Catto *et al* and builds on this work through inclusion of additional studies found through a systematic review and meta-analysis to synthesise the evidence across the studies.

The direction and magnitude of the reported impact of improved oxygen systems on child pneumonia mortality was similar across all four included studies despite variation in intervention design and delivery. A previous mixed-methods review of oxygen systems for paediatric care identified key features that contribute to practice change and sustainability, emphasising the importance of multidisciplinary team-based approaches that address both oxygen supply issues and how oxygen is used.^18^ While the four included studies in our review varied in strategy, they were all exemplars in this multidisciplinary and systematic approach and we recommend reading the individual study papers to learn more about what works in different contexts.^11–14 21–23^

**Figure 4:**
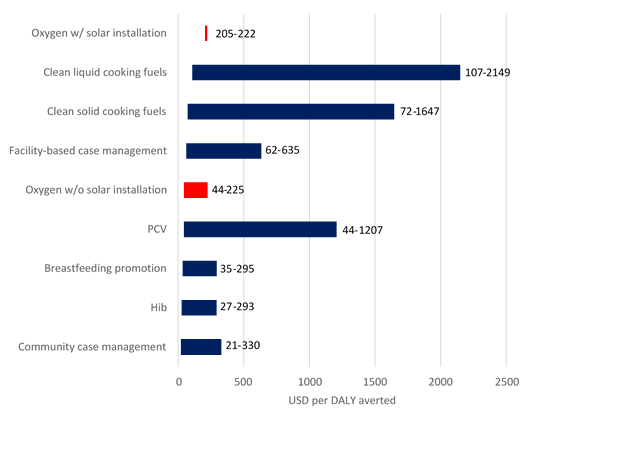
Cost-effectiveness of strengthening oxygen systems (with and without solar) presented alongside other child pneumonia interventions* (in USD in year 2000). *Cost-effectiveness estimates for other child pneumonia interventions were reproduced from Niessen *et al*.^24^ PCV, pneumococcal conjugate vaccine; Hib, Haemophilus influenza (H influenzae) type b vaccine.

Our cost-effectiveness analysis suggests that investments in strengthening oxygen systems are as cost-effective as other prioritised interventions such as vaccines, breastfeeding and indoor air pollution. Figure 4 depicts these results alongside cost-effectiveness results of other child pneumonia interventions found in an analysis conducted by Niessen *et al*.^24^ The cost-effectiveness analysis is likely a conservative estimate on the returns on investments to oxygen systems as we included all costs but limited effect calculations to children 1–59 months with pneumonia, for whom the best data on effectiveness exists, over relatively short study periods. However, oxygen systems in all participating facilities served a much broader population, including children with other illnesses, neonates and in some cases adult obstetric and general patients. When we examined cost-effectiveness for all-cause mortality among paediatric admissions—though the evidence was limited to Papua New Guinea—the cost per DALY averted fell by more than half. Our cost-effectiveness calculations were also restricted to the study periods (2–3 years), but we would expect these systems to continue working for at least 5 years with proper maintenance.^25^ A modelling analysis conducted by Huang *et al* estimated the cost-effectiveness of solar-powered oxygen systems over a 10-year period and found a cost-effectiveness estimate of US$20 per DALY averted.^26^ The included studies all used facility-based oxygen system solutions based on oxygen concentrators. While this fitted the clinical quality improvement approach of these small to medium-scale programmes, there are opportunities for increased efficiency by larger scale oxygen systems interventions that include a mix of oxygen supply technologies, policy and market shaping activities, and coordinated supply and distribution mechanisms. For example, while oxygen concentrators have utility in rapid deployment and rural settings, larger scale oxygen production and delivery methods, such as pressure swing adsorption plants and liquid oxygen can provide larger volumes of oxygen at a lower per unit cost and are likely to be more cost-efficient if combined with effective demand forecasting and distribution systems.

Our review was limited by the number and quality of the studies. Only four studies examining the effectiveness of strengthening oxygen systems were found during the search with one study conducted in Nigeria, one in Laos and two in Papua New Guinea. Three of the studies used a before-and-after design, and while Graham *et al* used a stepped-wedge cluster-randomised design, comparison against the preintervention period used a before-and-after approach. While we attempted to isolate the effect on children 1–59 months of age admitted to the facilities with a diagnosis of pneumonia, one of the studies (Duke) did not have age-disaggregated data for paediatric pneumonia admissions. The study author indicated that the vast majority (>90%) of paediatric pneumonia admissions were under-5 (personal correspondence Duke).

Using the GRADE framework, we assess the certainty in the evidence as low—meaning that further research is very likely to have an impact on our confidence in the estimate and change it. The reason for the low rating is primarily due to the observational design of the studies as we had no serious concerns about other characteristics of the study. However, it would be challenging to conduct an individually-randomised trial of oxygen therapy today for ethical reasons. Evidence for the clinical efficacy of oxygen as a medical therapy was established before clinical trials were developed, led by the work of John Scott Haldane and military medics during the first and second World Wars.^27–29^ As a result, oxygen therapy for treatment of hypoxaemia is standard of care and recommended by leading normative organisations such as WHO.^30^ Thus, withholding oxygen therapy from hypoxaemic children currently recommended to receive oxygen (ie, a blood oxygen saturation (SpO2) <90%) in order to estimate its clinical efficacy is likely to face ethical challenges. One recent multicentred trial attempted to examine the effect of different oxygen delivery strategies on mortality, including a control arm where children did not receive oxygen unless SpO_2_ was <80%0.^31^ The trial was halted by its steering committee before reaching its sample size as the study did not have sufficient funds to continue due to multiple study delays, one of which was a lawsuit over the legality and ethics of the trial.^32^

Importantly, the studies we reviewed were all assessing the impact of oxygen systems improvement programmes in facilities that lacked oxygen or had very limited access—not the clinical efficacy of oxygen as a medical therapy. Further programme implementation trials using rigorous study designs will continue to be important to generate evidence on successful implementation models, explore the use of oxygen in other settings such as outpatient and emergency referral, shine light on technical, clinical, economic and policy challenges, and contribute to the evidence base on mortality effects.^33–37^ Despite its ethical challenges, there also remain important areas of research regarding the clinical use of oxygen, including appropriate SpO2 thresholds for prescribing oxygen for different patient groups, health system contexts and geographical altitudes.^31 38^

Taking into consideration the review findings, we recommend including oxygen therapy as an intervention in LiST and provisionally using the pooled effect estimate and confidence intervals found in this review for the intervention effect and uncertainty parameters in LiST. The process and results of our review followed the intervention review standards for use in LiST described by the Child Health Epidemiology Reference Group (CHERG), and though the certainty of the effect estimate is ‘low’, this does not automatically preclude the intervention from being included in LiST.^39^ The CHERG guidelines recommend review of interventions graded as ‘low’ be included in the model but the intervention effect size should continue to be studied and as new evidence emerges that changes the effect estimate for oxygen, the parameters in LiST should be updated to reflect the best available evidence. Future research and discussion are also needed to define and measure oxygen therapy coverage to populate LiST coverage estimates.

## Conclusions

Strengthening oxygen systems in LMICs appears to reduce hospital-based pneumonia mortality rates in children under-5 and may be cost-effective. Additional implementation studies using more rigorous designs are needed to strengthen the certainty in the effect estimate.

## Data Availability

All data relevant to the study are included in the article or uploaded as online supplemental information. Not applicable.
