# Supplementary material for: Oxygen systems strengthening as an intervention to prevent childhood deaths due to pneumonia in low-resource settings: systematic review, meta-analysis and cost-effectiveness
Source: BMJ Glob Health. 2021 Dec 20;6(12):e007468. doi: 10.1136/bmjgh-2021-007468 (PMC8689120; doi:10.1136/bmjgh-2021-007468)
Supplement: Supplementary data [file bmjgh-2021-007468supp001.pdf]

Search terms

Concept #1

Pneumonia[Mesh:NoExp] OR hypoxia [mesh] OR Respiratory Tract Infections[Mesh:NoExp] OR **Oximetry[mesh]** OR Oxygen Deficienc*[tw] OR respiratory infection*[tw] OR Lung Inflammation*[tw] OR pneumonia*[tw] OR Pulmonary Inflammation*[tw] OR Hypoxemia[tw] OR Anoxia[tw] OR Anoxemia[tw] OR **Pulse Oximetr*[tw] OR Oximetr*[tw]**

Concept #2

Oxygen Inhalation Therapy[mesh] OR Oxygen Inhalation Therap*[tw] OR Respiratory Therapy[mesh] OR Respiratory Therap*[tw] OR Inhalation Therap*[tw]

Concept #3

Child, Preschool[mesh] OR Infant[mesh] OR Infant, Newborn[mesh] OR Preschool Child*[tw] OR infant*[tw] OR Newborn Infant*[tw] OR Newborn*[tw] OR Neonate*[tw]

Concept #4

afghanistan[tw] OR albania[tw] OR algeria[tw] OR american samoa[tw] OR angola[tw] OR antigua[tw] OR barbuda[tw] OR argentina[tw] OR armenia[tw] OR armenian[tw] OR aruba[tw] OR azerbaijan[tw] OR bahrain[tw] OR **bangladesh[tw]** OR barbados[tw] OR belarus[tw] OR byelarus[tw] OR belorussia[tw] OR byelorussian[tw] OR belize[tw] OR british honduras[tw] OR benin[tw] OR dahomey[tw] OR bhutan[tw] OR bolivia[tw] OR bosnia[tw] OR herzegovina[tw] OR botswana[tw] OR bechuanaland[tw] OR brazil[tw] OR brasil[tw] OR bulgaria[tw] OR burkina faso[tw] OR burkina fasso[tw] OR upper volta[tw] OR burundi[tw] OR urundi[tw] OR cabo verde[tw] OR cape verde[tw] OR cambodia[tw] OR kampuchea[tw] OR khmer republic[tw] OR cameroon[tw] OR cameron[tw] OR cameroun[tw] OR central african republic[tw] OR ubangi shari[tw] OR chad[tw] OR chile[tw] OR china[tw] OR colombia[tw] OR comoros[tw] OR comoro islands[tw] OR mayotte[tw] OR congo[tw] OR zaire[tw] OR costa rica[tw] OR cote d'ivoire[tw] OR ivory coast[tw] OR croatia[tw] OR cuba[tw] OR cyprus[tw] OR czech republic[tw] OR czechoslovakia[tw] OR djibouti[tw] OR french somaliland[tw] OR dominica[tw] OR dominican republic[tw] OR ecuador[tw] OR egypt[tw] OR united arab republic[tw] OR el salvador[tw] OR equatorial guinea[tw] OR spanish guinea[tw] OR eritrea[tw] OR estonia[tw] OR eswatini[tw] OR swaziland[tw] OR ethiopia[tw] OR fiji[tw] OR gabon[tw] OR gabonese republic[tw] OR gambia[tw] OR georgia[tw] OR georgian[tw] OR ghana[tw] OR gold coast[tw] OR gibraltar[tw] OR greece[tw] OR grenada[tw] OR guam[tw] OR **guatemala[tw]** OR guinea[tw] OR guyana[tw] OR guiana[tw] OR haiti[tw] OR hispaniola[tw] OR honduras[tw] OR hungary[tw] OR **india[tw]** OR indonesia[tw] OR timor[tw] OR iran[tw] OR iraq[tw] OR isle of man[tw] OR jamaica[tw] OR jordan[tw] OR kazakhstan[tw] OR kazakh[tw] OR **kenya[tw]** OR korea[tw] OR kosovo[tw] OR kyrgyzstan[tw] OR kirghizia[tw] OR kirgizstan[tw] OR kyrgyz republic[tw] OR kirghiz[tw] OR laos[tw] OR lao pdr[tw] OR lao people's democratic republic[tw] OR latvia[tw] OR lebanon[tw] OR lesotho[tw] OR basutoland[tw] OR liberia[tw] OR libya[tw] OR libyan arab jamahiriya[tw] OR lithuania[tw] OR macau[tw] OR macao[tw] OR macedonia[tw] OR madagascar[tw] OR malagasy republic[tw] OR malawi[tw] OR nyasaland[tw] OR malaysia[tw] OR maldives[tw] OR indian ocean[tw] OR mali[tw] OR malta[tw] OR micronesia[tw] OR kiribati[tw] OR marshall islands[tw] OR nauru[tw] OR northern mariana islands[tw] OR palau[tw] OR tuvalu[tw] OR mauritania[tw] OR mauritius[tw] OR mexico[tw] OR moldova[tw] OR moldovian[tw] OR mongolia[tw] OR

montenegro[tw] OR morocco[tw] OR ifni[tw] OR mozambique[tw] OR portuguese east africa[tw] OR myanmar[tw] OR burma[tw] OR namibia[tw] OR nepal[tw] OR netherlands antilles[tw] OR nicaragua[tw] OR niger[tw] OR nigeria[tw] OR oman[tw] OR muscat[tw] OR pakistan[tw] OR panama[tw] OR papua new guinea[tw] OR paraguay[tw] OR **peru[tw]** OR philippines[tw] OR philipines[tw] OR phillipines[tw] OR phillippines[tw] OR poland[tw] OR polish people's republic[tw] OR portugal[tw] OR portuguese republic[tw] OR puerto rico[tw] OR romania[tw] OR russia[tw] OR russian federation[tw] OR ussr[tw] OR soviet union[tw] OR union of soviet socialist republics[tw] OR rwanda[tw] OR ruanda[tw] OR samoa[tw] OR pacific islands[tw] OR polynesia[tw] OR samoan islands[tw] OR sao tome and principe[tw] OR saudi arabia[tw] OR senegal[tw] OR serbia[tw] OR seychelles[tw] OR sierra leone[tw] OR slovakia[tw] OR slovak republic[tw] OR slovenia[tw] OR melanesia[tw] OR solomon island[tw] OR solomon islands[tw] OR norfolk island[tw] OR somalia[tw] OR south africa[tw] OR south sudan[tw] OR sri lanka[tw] OR ceylon[tw] OR saint kitts and nevis[tw] OR st kitts and nevis[tw] OR saint lucia[tw] OR st lucia[tw] OR saint vincent[tw] OR st vincent[tw] OR grenadines[tw] OR sudan[tw] OR suriname[tw] OR surinam[tw] OR syria[tw] OR syrian arab republic[tw] OR tajikistan[tw] OR tadjikistan[tw] OR tadzhikistan[tw] OR tadzhik[tw] OR **tanzania[tw]** OR tanganyika[tw] OR thailand[tw] OR siam[tw] OR timor leste[tw] OR east timor[tw] OR togo[tw] OR togolese republic[tw] OR tonga[tw] OR trinidad[tw] OR tobago[tw] OR tunisia[tw] OR turkey[tw] OR turkmenistan[tw] OR turkmen[tw] OR **uganda[tw]** OR ukraine[tw] OR uruguay[tw] OR uzbekistan[tw] OR uzbek[tw] OR vanuatu[tw] OR new hebrides[tw] OR venezuela[tw] OR vietnam[tw] OR viet nam[tw] OR middle east[tw] OR west bank[tw] OR gaza[tw] OR palestine[tw] OR yemen[tw] OR yugoslavia[tw] OR zambia[tw] OR zimbabwe[tw] OR northern rhodesia[tw] OR global south[tw] OR africa south of the sahara[tw] OR sub saharan africa[tw] OR subsaharan africa[tw] OR central africa[tw] OR north africa[tw] OR northern africa[tw] OR magreb[tw] OR maghrib[tw] OR sahara[tw] OR southern africa[tw] OR east africa[tw] OR eastern africa[tw] OR west africa[tw] OR western africa[tw] OR west indies[tw] OR indian ocean islands[tw] OR caribbean[tw] OR central america[tw] OR latin america[tw] OR south america[tw] OR central asia[tw] OR north asia[tw] OR northern asia[tw] OR southeastern asia[tw] OR south eastern asia[tw] OR southeast asia[tw] OR south east asia[tw] OR western asia[tw] OR east europe[tw] OR eastern europe[tw] OR developing country[tw] OR developing countries[tw] OR developing nation[tw] OR developing nations[tw] OR developing population[tw] OR developing populations[tw] OR developing world[tw] OR less developed country[tw] OR less developed countries[tw] OR less developed nation[tw] OR less developed nations[tw] OR less developed world[tw] OR lesser developed countries[tw] OR lesser developed nations[tw] OR under developed country[tw] OR under developed countries[tw] OR under developed nations[tw] OR under developed world[tw] OR underdeveloped country[tw] OR underdeveloped countries[tw] OR underdeveloped nation[tw] OR underdeveloped nations[tw] OR underdeveloped population[tw] OR underdeveloped populations[tw] OR underdeveloped world[tw] OR middle income country[tw] OR middle income countries[tw] OR middle income nation[tw] OR middle income nations[tw] OR middle income population[tw] OR middle income populations[tw] OR **low income country[tw]** OR **low income countries[tw]** OR low income nation[tw] OR low income nations[tw] OR low income population[tw] OR low income populations[tw] OR lower income country[tw] OR lower income countries[tw] OR lower income nations[tw] OR lower income population[tw] OR lower income populations[tw] OR underserved countries[tw] OR underserved nations[tw] OR underserved population[tw] OR underserved populations[tw] OR under served population[tw] OR under served populations[tw] OR deprived countries[tw] OR deprived population[tw] OR deprived populations[tw] OR poor country[tw] OR poor countries[tw] OR poor nation[tw] OR poor nations[tw] OR poor population[tw] OR poor populations[tw] OR poor world[tw] OR poorer countries[tw] OR poorer nations[tw] OR poorer population[tw] OR poorer populations[tw] OR developing economy[tw] OR developing economies[tw] OR less developed economy[tw] OR less developed economies[tw] OR underdeveloped economies[tw] OR middle income economy[tw] OR middle income economies[tw] OR low income economy[tw] OR low income

economies[tw] OR lower income economies[tw] OR low gdp[tw] OR low gnp[tw] OR low gross domestic[tw] OR low gross national[tw] OR lower gdp[tw] OR lower gross domestic[tw] OR **lmic[tw]** OR lmics[tw] OR third world[tw] OR lami country[tw] OR lami countries[tw] OR transitional country[tw] OR transitional countries[tw] OR emerging economies[tw] OR emerging nation[tw] OR emerging nations[tw] OR afghanistan[mh] OR albania[mh] OR algeria[mh] OR american samoa[mh] OR angola[mh] OR antigua and barbuda[mh] OR argentina[mh] OR armenia[mh] OR aruba[mh] OR azerbaijan[mh] OR bahrain[mh] OR **bangladesh[mh]** OR barbados[mh] OR republic of belarus[mh] OR belize[mh] OR benin[mh] OR bhutan[mh] OR bolivia[mh] OR bosnia and herzegovina[mh] OR botswana[mh] OR brazil[mh] OR bulgaria[mh] OR burkina faso[mh] OR burundi[mh] OR cabo verde[mh] OR cambodia[mh] OR cameroon[mh] OR central african republic[mh] OR chad[mh] OR chile[mh] OR china[mh] OR colombia[mh] OR comoros[mh] OR democratic republic of the congo[mh] OR congo[mh] OR costa rica[mh] OR cote d'ivoire[mh] OR croatia[mh] OR cuba[mh] OR cyprus[mh] OR czech republic[mh] OR djibouti[mh] OR dominica[mh] OR dominican republic[mh] OR ecuador[mh] OR egypt[mh] OR el salvador[mh] OR equatorial guinea[mh] OR eritrea[mh] OR estonia[mh] OR eswatini[mh] OR ethiopia[mh] OR fiji[mh] OR gabon[mh] OR gambia[mh] OR "georgia republic"[mh] OR ghana[mh] OR gibraltar[mh] OR greece[mh] OR grenada[mh] OR guam[mh] OR **guatemala[mh]** OR guinea[mh] OR guinea-bissau[mh] OR guyana[mh] OR haiti[mh] OR honduras[mh] OR hungary[mh] OR **india[mh]** OR indonesia[mh] OR iran[mh] OR iraq[mh] OR jamaica[mh] OR jordan[mh] OR kazakhstan[mh] OR **kenya[mh]** OR democratic people's republic of korea[mh] OR republic of korea[mh] OR kosovo[mh] OR kyrgyzstan[mh] OR laos[mh] OR latvia[mh] OR lebanon[mh] OR lesotho[mh] OR liberia[mh] OR libya[mh] OR lithuania[mh] OR macau[mh] OR republic of north macedonia[mh] OR madagascar[mh] OR malawi[mh] OR malaysia[mh] OR indian ocean islands[mh] OR mali[mh] OR malta[mh] OR micronesia[mh] OR palau[mh] OR mauritania[mh] OR mauritius[mh] OR mexico[mh] OR moldova[mh] OR mongolia[mh] OR montenegro[mh] OR morocco[mh] OR mozambique[mh] OR myanmar[mh] OR namibia[mh] OR nepal[mh] OR netherlands antilles[mh] OR nicaragua[mh] OR niger[mh] OR nigeria[mh] OR oman[mh] OR pakistan[mh] OR panama[mh] OR papua new guinea[mh] OR paraguay[mh] OR **peru[mh]** OR philippines[mh] OR poland[mh] OR portugal[mh] OR puerto rico[mh] OR romania[mh] OR russia[mh] OR rwanda[mh] OR samoa[mh] OR sao tome and principe[mh] OR saudi arabia[mh] OR senegal[mh] OR serbia[mh] OR seychelles[mh] OR sierra leone[mh] OR slovakia[mh] OR slovenia[mh] OR melanesia[mh] OR somalia[mh] OR south africa[mh] OR south sudan[mh] OR sri lanka[mh] OR saint kitts and nevis[mh] OR saint lucia[mh] OR saint vincent and the grenadines[mh] OR sudan[mh] OR suriname[mh] OR syria[mh] OR tajikistan[mh] OR **tanzania[mh]** OR thailand[mh] OR timor-leste[mh] OR togo[mh] OR tonga[mh] OR trinidad and tobago[mh] OR tunisia[mh] OR turkey[mh] OR turkmenistan[mh] OR **uganda[mh]** OR ukraine[mh] OR uruguay[mh] OR uzbekistan[mh] OR vanuatu[mh] OR venezuela[mh] OR vietnam[mh] OR middle east[mh] OR yemen[mh] OR yugoslavia[mh] OR zambia[mh] OR zimbabwe[mh] OR africa south of the sahara[mh] OR africa, central[mh] OR africa, northern[mh] OR africa, southern[mh] OR africa, eastern[mh] OR africa, western[mh] OR west indies[mh] OR indian ocean islands[mh] OR caribbean region[mh] OR central america[mh] OR latin america[mh] OR south america[mh] OR asia, central[mh] OR asia, northern[mh] OR asia, southeastern[mh] OR asia, western[mh] OR europe, eastern[mh] OR **developing countries[mh]**
