# Supplementary material for: Oxygen systems strengthening as an intervention to prevent childhood deaths due to pneumonia in low-resource settings: systematic review, meta-analysis and cost-effectiveness
Source: BMJ Glob Health. 2021 Dec 20;6(12):e007468. doi: 10.1136/bmjgh-2021-007468 (PMC8689120; doi:10.1136/bmjgh-2021-007468)
Supplement: Supplementary data [file bmjgh-2021-007468supp002.pdf]

	Duke 2008	
**COMPONENT RATINGS**	**Rating**	**Explanation**
**(A) Selection bias**		
(Q1) Are the individuals selected to participate in the study likely to be representative of the target population?	Somewhat likely	Five hospitals were purposely selected but represented both highland and lowland areas. Page 2.
(Q2) What percentage of selected individuals agreed to participate?	60 – 79% agreement	All pneumonia admissions were included through retrospective register reviews though some of the registers were not available. Page 3.
**(B) Study design**		
(Q1) Indicate the study design	Cohort analytic (two group pre + post)	Pre + Post in the same facilities.
(Q2) Was the study described as randomized? If NO, go to Component C.	No	
(Q3) If Yes, was the method of randomization described? (See dictionary)		
(Q4) If Yes, was the method appropriate? (See dictionary)		
**(C) Confounders**		
(Q1) Were there important differences between groups prior to the intervention?<br>The following are examples of confounders:<br>1 Race<br>2 Sex<br>3 Marital status/family<br>4 Age<br>5 SES (income or class)<br>6 Education<br>7 Health status<br>8 Pre-intervention score on outcome measure	Can't tell	No description of patient demographics presented.
(Q2) If yes, indicate the percentage of relevant confounders that were controlled (either in the design (e.g. stratification, matching) or analysis)?	Can't tell	
**(D) Blinding**		
(Q1) Was (were) the outcome assessor(s) aware of the intervention or exposure status of participants?	Can't tell	Registers are filled by the hospital staff who knew the assessment plan but did not know the outcome data were coming from admission records books. Page 3-4.
(Q2) Were the study participants aware of the research question?	No	Unlikely as individual consent not sought and data collection relied on retrospective register reviews.
**(E) Data collection methods**		
(Q1) Were data collection tools shown to be valid?	Yes	Nursing staff in Papua New Guinea ensured that admission record books were accurate and up to date. Page 4.
(Q2) Were data collection tools shown to be reliable?	No	Several missing registers. Page 4.
**(F) Withdrawals and drop outs**		
(Q1) Were withdrawals and drop-outs reported in terms of numbers and/or reasons per group?	Yes	Several register books missing. Page 4.
(Q2) Indicate the percentage of participants completing the study. (If the percentage differs by groups, record the lowest).	60 - 79%	Out of a total of 380 hospital-months worth of registers, the study reviewed 302 (79%). Page 4.
**(G) Intervention integrity**		
(Q1) What percentage of participants received the allocated intervention or exposure of interest?	Can't tell	No data collected on intervention integrity.
(Q2) Was the consistency of the intervention measured?	No	
(Q3) Is it likely that subjects received an unintended intervention (contamination or co-intervention) that may influence the results?	No	
**(H) Analyses**		

(Q1) Indicate the unit of allocation (circle one)	organization/institution	Hospitals were the unit of allocation for the intervention.
(Q2) Indicate the unit of analysis (circle one)	organization/institution	Summary data of mortality rates at hospital collected.
(Q3) Are the statistical methods appropriate for the study design?	Yes	
(Q4) Is the analysis performed by intervention allocation status (i.e. intention to treat) rather than the actual intervention received?	Yes	
**GLOBAL RATINGS**		
**Component ratings**		
(A) Selection bias	Moderate	
(B) Study design	Moderate	
(C) Confounders	Weak	
(D) Blinding	Moderate	
(E) Data collection methods	Moderate	
(F) Withdrawals and drop outs	Moderate	
Global rating	Moderate (one weak rating)	

COMPONENT RATINGS	Gray 2017 Rating	Explanation
**(A) Selection bias**		
(Q1) Are the individuals selected to participate in the study likely to be representative of the target population?	Somewhat likely	Hospitals were not randomly selected but were purposely selected based on several criteria including infrastructure and case load. Hospitals across several geographies in LPDR were selected and within the hospitals, all pneumonia patients during the study period were included. Page 2.
(Q2) What percentage of selected individuals agreed to participate?	80 - 100% agreement	All pneumonia admissions at the hospitals were included through retrospective register reviews. Page 3.
**(B) Study design**		
(Q1) Indicate the study design	Cohort analytic (two group pre + post)	Pre + post with control and intervention facilities though analysis focuses on pre + post at the intervention facilities alone.
(Q2) Was the study described as randomized? If NO, go to Component C.	No	
(Q3) If Yes, was the method of randomization described? (See dictionary)		
(Q4) If Yes, was the method appropriate? (See dictionary)		
**(C) Confounders**		
(Q1) Were there important differences between groups prior to the intervention?<br>The following are examples of confounders:<br>1 Race<br>2 Sex<br>3 Marital status/family<br>4 Age<br>5 SES (income or class)<br>6 Education<br>7 Health status<br>8 Pre-intervention score on outcome measure	Yes	Patient numbers, age, length of stay and pneumonia severity were similar between hospital cohorts both preintervention and postintervention. There was a reduction in the proportion of severe/very severe pneumonia cases in the postintervention era. Page 5.
(Q2) If yes, indicate the percentage of relevant confounders that were controlled (either in the design (e.g. stratification, matching) or analysis)?	Can't tell	Analysis stratified by severity of pneumonia but did not discuss any other methods used to account for confounders.
**(D) Blinding**		
(Q1) Was (were) the outcome assessor(s) aware of the intervention or exposure status of participants?	Can't tell	Data were collected by a paediatric resident or paediatrician familiar with WHO classification of pneumonia and trained to identify signs or symptoms of pneumonia from records. Page 3.<br><br>Likely that data collectors knew that facility had received oxygen intervention or not but not explicitly addressed.
(Q2) Were the study participants aware of the research question?	No	Unlikely as individual consent not sought and data collection relied on retrospective case note reviews.
**(E) Data collection methods**		
(Q1) Were data collection tools shown to be valid?	Yes	Case note reviews seem to be valid and reliable and data collectors were trained pediatricians.
(Q2) Were data collection tools shown to be reliable?	No	The accuracy and detail of medical records limited our ability to accurately determine pneumonia severity or patient outcomes in some instances. Medical records represented those we could locate; we cannot be sure how many children were admitted with pneumonia in the 20 hospitals in each of the two time periods. Page 8.
**(F) Withdrawals and drop outs**		
(Q1) Were withdrawals and drop-outs reported in terms of numbers and/or reasons per group?	Can't tell	The accuracy and detail of medical records limited our ability to accurately determine pneumonia severity or patient outcomes in some instances. Medical records represented those we could locate; we cannot be sure how many children were admitted with pneumonia in the 20 hospitals in each of the two time periods. Page 8.

(Q2) Indicate the percentage of participants completing the study. (If the percentage differs by groups, record the lowest).	Not applicable (i.e. Retrospective case-control)	Data collection method used retrospective case note reviews.
**(G) Intervention integrity**		
(Q1) What percentage of participants received the allocated intervention or exposure of interest?	60 – 79% (some)	Documentation of SpO2 and O2 administration increased in the intervention arm but overall was still quite low. Less than 40% for documentation of SpO2 on all pneumonia and <50% for documentation of oxygen for hypoxemic patients. This may not reflect true intervention exposure but overall figures are low. Pages 6-7.
(Q2) Was the consistency of the intervention measured?	No	
(Q3) Is it likely that subjects received an unintended intervention (contamination or co-intervention) that may influence the results?	No	
**(H) Analyses**		
(Q1) Indicate the unit of allocation (circle one)	organization/institution	Hospitals were selected to receive the intervention.
(Q2) Indicate the unit of analysis (circle one)	organization/institution	Summary data of mortality rates at hospital collected. Some individual data collected on severity of disease and age but outcome data was aggregated at facility level for analysis.
(Q3) Are the statistical methods appropriate for the study design?	Yes	
(Q4) Is the analysis performed by intervention allocation status (i.e. intention to treat) rather than the actual intervention received?	Yes	All pneumonia patients in post-intervention arm included in the analysis (not only the ones that received oxygen).
**GLOBAL RATINGS**		
**Component ratings**		
(A) Selection bias	Moderate	
(B) Study design	Moderate	
(C) Confounders	Weak	
(D) Blinding	Moderate	
(E) Data collection methods	Moderate	
(F) Withdrawals and drop outs	Moderate	
Global rating	Moderate (one weak rating)	

	Graham 2019	
**COMPONENT RATINGS**	**Rating**	**Explanation**
**(A) Selection bias**		
(Q1) Are the individuals selected to participate in the study likely to be representative of the target population?	Somewhat likely	Hospitals purposely selected in collaboration with state health authorities to be representative of secondary-level health facilities that provide inpatient pediatric care with at least 150 admissions per years. Govt and state-run hospitals included only. Four states in SW Nigeria (Oyo, Ondo, Ogun, and Osun).
(Q2) What percentage of selected individuals agreed to participate?	80 - 100% agreement	Dat on all children <15 that were admitted to the hospital were included.
**(B) Study design**		
(Q1) Indicate the study design	Randomized controlled trial	RCT for comparing pulse oximetry vs full oxygen support interventions. Pre + post cohort study for no intervention vs full oxygen support.
(Q2) Was the study described as randomized? If NO, go to Component C.	Yes	
(Q3) If Yes, was the method of randomization described? (See dictionary)	Yes	Page 6.
(Q4) If Yes, was the method appropriate? (See dictionary)	Yes	
**(C) Confounders**		
(Q1) Were there important differences between groups prior to the intervention?<br>The following are examples of confounders:<br>1 Race<br>2 Sex<br>3 Marital status/family<br>4 Age<br>5 SES (income or class)<br>6 Education<br>7 Health status<br>8 Pre-intervention score on outcome measure	Yes	Changes in admission patterns between pre + post.
(Q2) If yes, indicate the percentage of relevant confounders that were controlled (either in the design (e.g. stratification, matching) or analysis)?	80 – 100% (most)	'severity-adjusted' analysis including individual signs of illness severity, age, sex, and type ofhospital (mission versus government) as additional fixed effects. Pages 8-9.
**(D) Blinding**		
(Q1) Was (were) the outcome assessor(s) aware of the intervention or exposure status of participants?	Can't tell	Outcomes were extracted from caes notes by data collectors based at hospital but employed by study. Likely data collectors knew when intervention introduced to facility but not explicitly addressed.
(Q2) Were the study participants aware of the research question?	No	Unlikely as individual consent not sought and data collection relied on retrospective case note reviews.
**(E) Data collection methods**		
(Q1) Were data collection tools shown to be valid?	Yes	Our use ofdedicated research nurses minimised the amount ofmissing data, and our audit ofdocumentation practices prior to starting the study reassured us that documentation practices overall were excellent [13]. Page 18.
(Q2) Were data collection tools shown to be reliable?	Yes	As above.
**(F) Withdrawals and drop outs**		
(Q1) Were withdrawals and drop-outs reported in terms of numbers and/or reasons per group?	Can't tell	Data collection method used retrospective case note reviews.
(Q2) Indicate the percentage of participants completing the study. (If the percentage differs by groups, record the lowest).	Not applicable (i.e. Retrospective case-control)	
**(G) Intervention integrity**		
(Q1) What percentage of participants received the allocated intervention or exposure of interest?	80 – 100% (most)	
(Q2) Was the consistency of the intervention measured?	No	

(Q3) Is it likely that subjects received an unintended intervention (contamination or co-intervention) that may influence the results?	No	
**(H) Analyses**		
(Q1) Indicate the unit of allocation (circle one)	organization/institution	Hospitals were randomized to intervention phasing.
(Q2) Indicate the unit of analysis (circle one)	individual	Individual patient data used in the modeling analysis.
(Q3) Are the statistical methods appropriate for the study design?	Yes	
(Q4) Is the analysis performed by intervention allocation status (i.e. intention to treat) rather than the actual intervention received?	Yes	Page 8
**GLOBAL RATINGS**		
**Component ratings**		
(A) Selection bias	Moderate	
(B) Study design	Moderate	The analysis of relevance for the systematic review uses the cohort analytic design (pre + post)
(C) Confounders	Strong	
(D) Blinding	Moderate	
(E) Data collection methods	Strong	
(F) Withdrawals and drop outs	Moderate	
Global rating	Strong (no weak ratings)	

	Duke 2020	
COMPONENT RATINGS	Rating	Explanation
**(A) Selection bias**		
(Q1) Are the individuals selected to participate in the study likely to be representative of the target population?	Somewhat likely	We selected health facilities on the basis of high community burden of pneumonia, lack of reliable source of oxygen, limited or unreliable power, and staff being committed and enthusiastic to participate. Page 2.
(Q2) What percentage of selected individuals agreed to participate?	Can't tell	Some data excluded due to low quality registers but not able to quantify. Overall seems like data were well kept as to not exclude patients. Pages 2-3.
**(B) Study design**		
(Q1) Indicate the study design	Cohort analytic (two group pre + post)	
(Q2) Was the study described as randomized? If NO, go to Component C.	No	
(Q3) If Yes, was the method of randomization described? (See dictionary)		
(Q4) If Yes, was the method appropriate? (See dictionary)		
**(C) Confounders**		
(Q1) Were there important differences between groups prior to the intervention?<br>The following are examples of confounders:<br>1 Race<br>2 Sex<br>3 Marital status/family<br>4 Age<br>5 SES (income or class)<br>6 Education<br>7 Health status<br>8 Pre-intervention score on outcome measure	Can't tell	Patient register books used so no individual data on patient demographics available to compare pre + post.
(Q2) If yes, indicate the percentage of relevant confounders that were controlled (either in the design (e.g. stratification, matching) or analysis)?	Can't tell	
**(D) Blinding**		
(Q1) Was (were) the outcome assessor(s) aware of the intervention or exposure status of participants?	Can't tell	Clinical staff knew about the evaluation of the solar oxygen system; however, they did not know that outcome data were derived from ward admission record books. The accuracy with which data were recorded did not change during the study. There were significantly fewer children with pneumonia admitted in the postintervention era, and this requires some consideration. Page 5.
(Q2) Were the study participants aware of the research question?	No	
**(E) Data collection methods**		
(Q1) Were data collection tools shown to be valid?	Yes	
(Q2) Were data collection tools shown to be reliable?	No	The data were collected from the health facility admission and discharge record books, which are generally kept meticulously by senior nursing staff in PNG. Each facility has a record book, and details of every admission is entered manually; the data include patient name, contact address, diagnosis at admission and discharge, and outcome. Page 2.<br><br>Some facilities and periods excluded from analysis due to low quality data.
**(F) Withdrawals and drop outs**		
(Q1) Were withdrawals and drop-outs reported in terms of numbers and/or reasons per group?	Can't tell	See above on case note reviews. Some facilities and periods excluded due to missing or low quality registers.
(Q2) Indicate the percentage of participants completing the study. (If the percentage differs by groups, record the lowest).	Not applicable (i.e. Retrospective case-control)	
**(G) Intervention integrity**		

(Q1) What percentage of participants received the allocated intervention or exposure of interest?	Can't tell	No data collected on intervention integrity.
(Q2) Was the consistency of the intervention measured?	No	
(Q3) Is it likely that subjects received an unintended intervention (contamination or co-intervention) that may influence the results?	No	
**(H) Analyses**		
(Q1) Indicate the unit of allocation (circle one)	organization/institution	Hospitals were the unit of allocation for the intervention.
(Q2) Indicate the unit of analysis (circle one)	organization/institution	Summary data of mortality rates at hospital collected.
(Q3) Are the statistical methods appropriate for the study design?	Yes	
(Q4) Is the analysis performed by intervention allocation status (i.e. intention to treat) rather than the actual intervention received?	Yes	
**GLOBAL RATINGS**		
**Component ratings**		
(A) Selection bias	Moderate	
(B) Study design	Moderate	
(C) Confounders	Weak	
(D) Blinding	Moderate	
(E) Data collection methods	Moderate	
(F) Withdrawals and drop outs	Moderate	
Global rating	Moderate (one weak rating)	
